# Spider Cancer

**Published:** 1875-05

**Authors:** 


### SPIDER CANCER.

What is commonly known as "spider cancer" is a very harmless thing. It is simply an enlargement and engorgement of several very small blood vessels, caused by some strain, as in violent coughing. This enlargement has a small central point, quite red, from which several small, but engorged veins, radiate, giving the shape of a small spider. It is often met with, and in persons of fair complexion, is quite noticeable, as it is generally located on the face, just below the eye. If left to itself, the tendency is to enlarge. It is, however, easily cured. With a sharp lancet prick through the skin at the nucleus, or central point. A drop or two of blood will ooze out, and the spot will at once disappear. When the bleeding has entirely stopped, insert into the wound for an instant the sharpened point of a stick of nitrate of silver. This destroys the little blood vessel and completes the cure.

---

Babies should not be allowed to suck their thumbs. It does serious injury in several ways. The habit induces protruding and bad shaped lips, and destroys the contour of the thumb; more than this, it seriously deforms the chest. The arm of a child addicted to this habit lies much of the time in only one position, and its weight on the thorax occasions a depression of the ribs, to the serious diminution of the lung-capacity. It is safe to predict that the constitution of a child who is indnlged in this practice will be likely to be seriously and permanently impaired.

---

Dust is chiefly organic vegetable matter. The greater part of the dust of city streets is horse manure, and the other offal. To inhale it is unquestionably destructive to human life.

